# Salivary Biomarker Analysis to Distinguish Between Health and Periodontitis Status: A Preliminary Study

**DOI:** 10.3390/dj13090436

**Published:** 2025-09-22

**Authors:** Carlo Bertoldi, Milena Nasi, Roberta Salvatori, Marcello Pinti, Silvia Montagna, Maurizio Tonetti, Luigi Generali, Elisa Bellei, Davide Zaffe, Valentina Selleri, Stefania Bergamini

**Affiliations:** 1 Department of Surgery, Medicine, Dentistry and Morphological Sciences with Transplant Surgery, Oncology and Regenerative Medicine Relevance, University of Modena and Reggio Emilia, 41124 Modena, MO, Italy; carlo.bertoldi@unimore.it (C.B.); milena.nasi@unimore.it (M.N.); luigi.generali@unimore.it (L.G.); elisa.bellei@unimore.it (E.B.); stefania.bergamini@unimore.it (S.B.); 2 Department of Medical and Surgical Sciences for Children and Adults, School of Medicine, University of Modena and Reggio Emilia, 41124 Modena, MO, Italy; 3 Department of Life Sciences, University of Modena and Reggio Emilia, Via G. Campi 287, 41125 Modena, MO, Italy; marcello.pinti@unimore.it (M.P.); valentina.selleri@unimore.it (V.S.); 4 Marco Biagi Department of Economics, University of Modena and Reggio Emilia, via Jacopo Berengario, 51, 41121 Modena, MO, Italy; silvia.montagna@unimore.it; 5 Perio-Implant Innovation Centre, Shanghai Ninth People’s Hospital, Shanghai Jiao Tong University School of Medicine, Shanghai 200023, China; maurizio.tonetti@shsmu.edu.cn; 6 College of Stomatology, Shanghai Jiao Tong University, Shanghai 200032, China; 7 Medical Clinical Research Centre of Shanghai Ninth People’s Hospital, Shanghai Jiao Tong University, Shanghai 200032, China; 8 European Research Group on Periodontology, 3855 Brienz Be, Switzerland; 9 Department of Biomedical, Metabolic and Neural Sciences, University of Modena and Reggio Emilia, 41125 Modena, MO, Italy; davide.zaffe@unimore.it

**Keywords:** periodontitis, periodontal therapy, periodontitis biomarker, matrix metalloproteinase, inflammation, cytokine

## Abstract

**Objective:** This study aims to explore the feasibility of a non-invasive and simple method for discriminating between health and periodontitis (PRD), facilitating early and objective diagnosis of PRD before detectable periodontal attachment loss and monitoring treatment outcomes. **Methods:** Salivary samples were collected from 16 PRD-free patients (G1) and 10 patients with PRD (G2). The analysis included salivary matrix metalloproteinase-8 (MMP-8), major anti-inflammatory interleukins (IL-4 and IL-10), pro-inflammatory cytokines (IL-1β, IL-8, and interferon α [IFN-α]), and the cytokine IL-6. Clinical and salivary assessments were performed at baseline (TP0) for both groups and after periodontal treatment for G2 (TP1). **Results:** PRD indices were significantly higher in G2-TP0, lower in G1, and intermediate in G2-TP1. Except for IL-6, the biomarkers were significantly correlated with nearly all PRD clinical indices. Logistic regression and receiver operating characteristic (ROC) curve analyses showed statistical significance for MMP-8, IL-1β, IL-4, IL-8, and IL-10 when comparing G1 and G2 at TP0. MMP-8 was also significant when comparing G2-TP0 and G2-TP1, while IL-1β and IL-10 showed borderline significance. IL-8 was significant when comparing G1 and G2-TP1. **Conclusions:** The molecular network demonstrated great potential for early diagnosis and monitoring of therapy response, providing a promising basis for future research. Among the biomarkers, MMP-8, IL-1β, IL-4, IL-8, and IL-10 showed the strongest statistical correlations with the clinical indices. The inflammation-related biomolecules behaved differently among untreated PRD (G2-TP0), treated (G2-TP1), and healthy individuals (G1). Healthy individuals and those with treated PRD may regulate inflammation significantly differently from those with untreated PRD.

## 1. Introduction

Periodontitis (PRD) is one of the most prevalent dysbiotic oral diseases triggered by microbial ecosystems. It involves severe chronic inflammation, clinical attachment loss, and alveolar bone resorption and can ultimately lead to tooth loss [1,2]. However, this pathogenic model does not fully capture the dynamic nature of biochemical processes, such as the innate differences among subjects and changes in environmental factors that may accelerate or dampen biochemical changes. A modern pathogenetic model, incorporating protein, cytokine, and metabolite data into dynamic biological processes, must be based on a multilevel framework that includes both disease-initiating and -resolving mechanisms, which are regulated by innate and environmental factors [3,4,5,6]. More recently, the role of cytokines and other protein mediators of inflammation and tissue damage has emerged as critical in PRD pathogenesis and, consequently, diagnosis [7,8,9,10,11,12,13,14,15,16].

Among the most important clinical discoveries of the last few decades is the involvement of the immune system and inflammatory processes in a wide range of health problems that dominate global morbidity and mortality [17,18].

Furthermore, chronic inflammatory diseases have been identified as the leading cause of over 50% of all deaths worldwide [19].

The risk of developing chronic inflammation is traced back to early development, with effects now known to persist throughout the lifespan, affecting adult health, causing disability, and even increasing the risk of mortality [20]. Many chronic diseases, such as PRD, share a common inflammatory and immune control pathogenesis, particularly in the transition from acute to chronic inflammation. These illnesses, including cardiovascular diseases (CVDs), type 2 diabetes mellitus (DM), rheumatoid arthritis (RA), cancer, autoimmune diseases, and bowel diseases, are associated with PRD [5,18,21,22,23,24,25,26]. Therefore, PRD is considered a systemically relevant disease and is the sixth most prevalent pathological condition in the world [27].

The major share of periodontal and systemic damage induced by PRD is attributed to chronic inflammation [18,28,29,30,31,32].

Despite what is already known, the diagnosis of PRD is still mainly established through clinical criteria. Periodontal attachment loss and the detection of periodontal pockets—clinical manifestations of PRD—are the primary clinical findings on which the diagnosis is based. This is due to the lack of reliable pathogenic criteria tied to a specific molecular network that is strictly and uniquely correlated with the disease [33,34,35].

The current gap in understanding the pathogenesis of PRD leads to three significant consequences. First, PRD diagnosis is based on the presence of established, clinically evident, and irreversible pathological changes (i.e., the damage is not preventable). Second, periodontal therapy is primarily targeted at dysbiotic ecosystems—those that trigger PRD—but does not address the host response or modify the periodontopathic inflammatory diathesis.

Third, the diagnosis should be carried out exclusively by experienced operators; otherwise, it becomes largely subjective, relying on periodontal probing due to the absence of a molecular network that could serve as an objective marker for PRD.

Salivary matrix metalloproteinase-8 (MMP-8) is currently considered a promising marker for both the diagnosis and treatment of PRD [14,15]. MMP-8 is a collagenase that cleaves extracellular matrix proteins, primarily type I, II, and III collagens, in physiological processes. However, MMP-8 is associated not only with PRD, but also with rheumatoid arthritis (RA), certain types of cancer, cancer progression, immune cell infiltration, connective tissue damage, smoking, obesity, and the regulation of the innate immune system [36,37,38]. Therefore, while MMP-8 shows promise as a marker for PRD, it does not specifically target periodontitis.

PRD tissue damage is most likely driven by the onset of acute inflammation, which fails to resolve, thus becoming chronic. Therefore, evaluating a set of molecules closely linked to PRD—molecules involved in the initiation and resolution of the inflammatory process—could help to characterize a reliable early diagnostic molecular network. Such a network could potentially anticipate the onset of periodontal damage and enhance our understanding of PRD pathogenesis.

By assessing the periodontal immuno-inflammatory network, the current diagnostic guidelines based on clinical parameters may improve their predictive ability. This pathway would be grounded in molecular network analysis, which would also provide additional benefits in terms of patient care, particularly by offering a minimally invasive method to facilitate early diagnosis. Thus, the aim of this study is to explore the feasibility of using the local molecular network to discriminate between health and PRD and use such information to develop and test a practical diagnostic tool in future studies.

## 2. Materials and Methods

This clinical study was performed at the Modena University Hospital, Periodontology Unit of Dentistry and Oral-Maxillofacial Surgery, 41124 Modena, Italy. All procedures were approved and supervised by the local ethical committee of the Health Service of the Emilia-Romagna region (University-Hospital of Modena- protocol nr 3968/2017, registration nr 315/17 and subsequent substantial amendment protocol AOU 0026796/22). Enrolled subjects signed informed consent detailing all procedures of the study, as requested by the Helsinki protocols [39].

Subjects referred for dental and periodontal evaluation were selected between January 2023 and February 2024.

### 2.1. Participants

Patients in good general health were screened. Inclusion criteria were as follows: 18 years or older, non-pregnant or lactating, non-smokers, no history of alcohol abuse, medical history of good health. Exclusion criteria comprised the following: bone disease, systemic or local inflammatory or infectious conditions (excluding periodontitis), use of anti-inflammatory drugs, uncontrolled or poorly controlled diabetes, unstable or life-threatening conditions, edentulous adults, use of antibiotics within the previous 3 months, and receipt of professional periodontal treatment (other than supragingival cleaning) within the previous 12 months [40,41]. This was a two-parallel-group study. The first group (G1) included healthy subjects who requested a dental visit without gingivitis or PRD [34,35]. The second group (G2) consisted of patients suffering from stage 3 or 4 PRD (severe PRD) [35]. The study was designed with two time points (TPs): baseline (TP0) and 6-month follow-up (TP1).

### 2.2. Clinical Measurements

Clinical measurements for periodontal charting were taken by an independent, trained, and experienced examiner (L.G.).

Periodontal charting was performed using a periodontal probe (PCP-UNC 15 tip, Hu-Friedy, Chicago, IL, USA), to the nearest millimeter, and radiological status was assessed when indicated [34,35].

Full-mouth plaque score (FMPS), assessed by plaque disclosing gel, was recorded as the percentage of total surfaces (six aspects per tooth) displaying the presence of plaque. Bleeding on probing (BoP) was assessed dichotomously using a periodontal probe with 1-millimeter marks (modified Click-Probe, Kerr Corp., Bioggio, Switzerland), and full-mouth bleeding score (FMBS) was then calculated (six aspects per tooth) [42].

Probing pocket depth (PPD) and gingival recession depth (REC) were recorded to the nearest millimeter. Clinical attachment level (CAL) was calculated as the sum of PPD and REC [4]. The indices PPD and REC were considered together in the respective full-mouth indices, as mean probing depth (MPD) and mean attachment level (MAL). The number of pockets with PPD ≥ 5 mm per patient (NPP5), representing pockets of moderate or greater severity [43], was also considered.

### 2.3. Periodontal Procedures

Dental and periodontal conditions were assessed and treated according to the individual needs and requirements of each patient.

The first phase aimed to guide behavior change by motivating the patient. It included patient evaluation, case history, and dental screening. Emergencies, such as pulpitis or dental abscess, were treated. At this stage, a distinction was made between subjects with periodontal health and those with severe PRD (Stage III/IV).

Afterwards, periodontal charting and periodontal diagnostic-quality radiographs (when appropriate) were performed at TP0, for both G1 and G2 patients. At TP1, these were performed only for G2 patients.

TP0 was considered the baseline. At TP0, patients were enrolled and signed the informed consent if they met all requirements.

Periodontal indices and salivary samples were taken at TP0 for both G1 and G2 patients, and at TP1 only for G2 patients.

The periodontal therapy, aimed to guide behavioral changes by motivating patients to effectively remove dental biofilm and control periodontal risk factors (by reducing or eliminating them) through supragingival and subgingival non-surgical periodontal therapy (NSPT), was performed by a single experienced periodontist (CB), in accordance with the Clinical Practice Guidelines [2,44,45,46].

**Figure 1:**
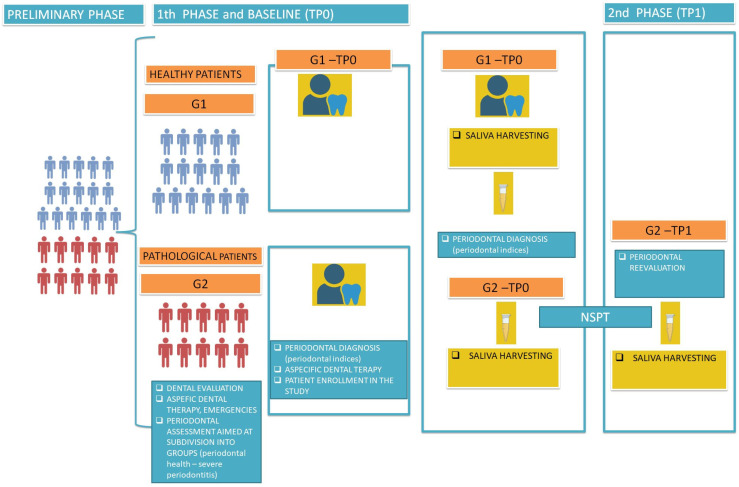
Workflow of the study. G1 included systemically healthy subjects, without gingivitis and periodontitis; G2 included systemically healthy subjects suffering from severe or advanced periodontitis. Legend: TP = time point. NSPT = non-surgical periodontal therapy.

After 180 days following the completion of the NSPT, which was only performed on G2 patients, an oral evaluation, complete with periodontal charting and salivary sample collection, was executed again at TP1 (Figure 1).

### 2.4. Salivary Sample Collection

Salivary samples were collected at baseline (TP0) and after periodontal treatment of G2 (TP1). The collection was performed by a SalivaBio oral saliva swab (Salimetrics, State College, PA, USA).

Subjects were required to avoid eating, consuming sugary drinks, fruit juices, carbonated beverages, coffee, or alcohol for at least 8 h. They were allowed to drink water when thirsty but were instructed to avoid excessive intake. Additionally, subjects had to refrain from drinking, brushing their teeth, or using mouthwash for at least 30 min prior to the test. The salivary collection was administered to the participants by a trained periodontist (CB), following the manufacturer’s instructions through the following steps: (a) a 30 s rinse with tap water; (b) a 60 s wait after the rinse; (c) intraoral positioning of proprietary and sterile salivary rollers; (d) collection of rollers in proprietary salivary storage tubes.

The samples were immediately centrifuged at 1500× *g* for a period of 15 min at +4 °C. The concentrated salivary samples were then aliquoted in sterile Eppendorf tubes and stored at −80 °C for further analysis.

### 2.5. Matrix Metalloproteinase-8 Protein (MMP-8) Assay

For MMP-8, quantification was performed using the Quantikine^®^ ELISA kit (DMP800B, Bio-Techne, Minneapolis, MN, USA), which includes eight standard concentrations (0, 0.156, 0.313, 0.625, 1.25, 2.5, 5, and 10 ng/mL). Using these standards, a four-parameter logistic (4PL) calibration curve was reconstructed to confirm the validity of the assay range.

The obtained absorbance values (OD at 540 nm) for all samples fell within the dynamic range defined by the standard curve, and none were below the average detection limit (0.013 ng/mL); therefore, all samples were considered accurate for analysis.

The MMP-8 Immunoassay (Quantikine^®^ELISA, Bio-Techne, Minneapolis, MN, USA) consisted an ELISA test used to measure total MMP-8 (pro and active MMP-8) in salivary and GCF samples. In the immunoassay, a monoclonal antibody specific for human MMP-8 was pre-coated on a microtiter plate. Fifty-microliter aliquots of standards and samples were aliquoted into the wells, and any MMP-8 present bound to the immobilized antibody. After washing away any unbound substances, an enzyme-linked monoclonal antibody specific for human MMP-8 was added to the wells. After washing to remove any unbound enzyme–antibody reagent, a substrate solution was added to the wells. The color in the wells changes from blue to yellow, producing a color proportional to the concentration of MMP-8 found in the salivary samples. The optical density of each well was measured using a microplate reader set to 540 nm [47,48].

### 2.6. Cytokine Immunological Assay

Cytokine concentrations were obtained using the Simple Plex™ ELLA platform (ProteinSimple), which automatically generates calibration curves within each cartridge, and all sample values were found to be above the respective detection thresholds.

Before the test, the samples were thawed and centrifuged at 4 °C for 15 min at 12,000 rpm. Salivary samples were diluted 1:2 in sample diluent (ProteinSimple). Fifty microliters of the mixture from each salivary sample were loaded into the multi-analyte cartridge for the simultaneous measurement of cytokines (IL-1β, IL-4, IL-6, IL-8, IL-10) and IFN-α using the automated platform “Simple Plex ELLA” (ProteinSimple, Bio-Techne, Minneapolis, MN, USA) [49,50].

All cytokine measurements were performed in duplicate using the same batch of reagents. Standard curves generated from known concentrations provided in the assay kits, along with a blank (zero control), were used for calibration. The intra-assay coefficients of variation were <10% for all analytes, indicating good reproducibility.

### 2.7. Study Outcomes

Primary outcomes included characterizing an effective local molecular network to distinguish between healthy individuals and those with PRD and using this information to develop and evaluate a practical diagnostic test in future studies.

The secondary outcomes were to obtain effective indications for PRD treatment and improve the pathogenic knowledge of PRD.

### 2.8. Statistical Analysis

Data were expressed as mean (M) ± standard deviation (SD), standard error (SE), and median to describe the sample distribution [51]. For all measured variables, comparisons between G1 and G2 (independent groups) were performed using the Wilcoxon rank-sum test, while comparisons within G2 (TP0 vs. TP1, paired data) were conducted using the Wilcoxon signed-rank test. The null hypothesis (H_0_) of no difference among groups was rejected at a critical significance level of *p* < 0.05.

Additionally, we examined the correlation between each clinical index–biomarker pair using Spearman’s rank correlation (*ρ*) separately for each group (G1, G2 at TP0, and G2 at TP1). For all tests, the null hypothesis (H_0_) of no significant correlation between a clinical variable and a biomarker was rejected at *p* < 0.05.

Finally, to analyze the biomarker network relevant to oral health, we performed a cluster analysis using the k-means algorithm to identify natural groupings of salivary biomarkers and clinical indices. Biomarkers and clinical indices grouped within the same cluster may share underlying biological relationships, suggesting they co-vary across individuals. This co-occurrence could indicate that certain biomarkers reflect or predict clinical measures of disease severity. We also applied linear regression (LR) analysis, fitting LR models separately for each group (G1, G2 at TP0, and G2 at TP1), with each clinical index as the outcome and all biomarkers as predictors. Categorical and continuous variables (mean ± SD), collected at TP0 and TP1, were coded and entered into a spreadsheet by a single blinded author (E.B.).

To investigate associations between the seven biomarkers and periodontal health status (healthy controls—G1; untreated PRD patients—G2 at TP0; PRD patients after treatment—G2 at TP1), we first fitted a multivariate logistic regression model with disease status as the outcome and all seven biomarkers as predictors. All covariates were standardized (mean-centered and scaled to unit variance) to facilitate coefficient interpretation and improve numerical stability.

Diagnostic testing using the “detect separation” package in R was conducted to assess model fit. If the results confirmed the presence of complete separation, indicating that one or more biomarkers perfectly distinguished between outcome groups, this resulted in unreliable or infinite coefficient estimates in the multivariate model. To address this issue while retaining interpretability, we conducted univariate logistic regression analyses for each standardized biomarker.

To assess classification performance, we performed ROC curve analysis using the “pROC” package in R. Each ROC curve represents the diagnostic ability of a single biomarker to distinguish between PRD cases (G2 at TP0), PRD after treatment (G2 at TP1), and healthy controls (G1), based on predicted probabilities from the corresponding univariate model. The area under the curve (AUC) quantifies overall classification accuracy, with higher AUC values indicating better performance. These ROC curves provide both visual and quantitative assessments of each biomarker’s potential utility as a standalone indicator of PRD.

All the statistical analyses were performed by a single blinded statistician (S.M.). Statistical analyses were performed with the pROC (version 1.19.0.1) [52], the qgraph package (version 1.9.8) [53] and the R software (version 4.5.1) [54].

## 3. Results

### 3.1. Examiner Calibration

In the pre-experimental period, the examiner’s calibration procedures were performed through a clinical periodontal evaluation of six participants. The periodontal parameters considered were obtained on two separate occasions (14-day intervals). Data were tabulated and submitted to the Spearman correlation coefficient test. The obtained values were 0.89 ÷ 0.99 (i.e., excellent concordance).

### 3.2. Descriptive Clinical Analysis

The study included 26 participants drawn from 32 consecutive potential patients. Of the 32 potential patients, two did not provide informed consent to participate in the study, three revealed they had taken antibiotics, and one did not attend the follow-up due to relocating for work in another city.

G1 consisted of 16 patients, aged 24.9 ± 4.53 years (range 18–32 years). All the patients had 28 teeth (wisdom teeth were excluded from the count). The FMPS and FMBS were 8.13 ± 6.03% (M ± SD) and 3.63 ± 3.12%, respectively. G1 patients were periodontally healthy (EFP), so the CAL was 0 in all cases. The mean probing depth (MPD) was 1.24 ± 0.15 mm. They did not show any periodontal pocket, and FMBS < 10%. G1 patients did not suffer from PRD or gingivitis; therefore, no specific periodontal treatment was implemented to treat these patients, so they were considered only at TP0.

G2 consisted of 10 non-smoker patients. G2 patients were 52.2 ± 8.28 years of age (range 43–66). G2 patients had 25.6 ± 4.30 teeth (wisdom teeth excluded) per patient and suffered from periodontitis stage 3 or 4 (five in the third and five in the fourth stage). In all cases, they showed grade C PRD.

At TP0, FMPS and FMBS were 41.10 ± 22.20% and 33.80 ± 15.34%, respectively. The MPD and MAL were 2.63 ± 0.65 mm and 3.56 ± 0.75 mm, respectively. G2 patients showed 190 pockets with PPD ≥ 5 mm, and their NPP5 was 19.00 ± 12.76. 

At TM1, FMPS and FMBS were 20.72 ± 9.68% and 16.90 ± 10.82%, respectively. The MPD and the MAL were 1.94 ± 0.42 mm and 2.95 ± 0.67 mm, respectively. At TP1, G2 patients had 73 pockets with PPD ≥ 5 mm, with an NPP5 value of 7.3 ± 11.12.

Both FMPS and FMBS were significantly higher in G2 patients at TP0 vs. G1 (*p* < 0.05) (Table 1). The Wilcoxon signed-rank test confirmed that both FMPS and FMBS were significantly higher in G2 patients at TP0 vs. G2 patients at TP1 (*p* = 0.025 and 0.014, respectively). Significant FMPS and FMBS mean reductions (20.38% and 16.9%, respectively) were observed between TP0 and TP1 for G2 patients. G1 patients showed significantly lower FMPS and FMBS than G2 patients at TP0 or TP1 (Table 1).

MPD and MAL were significantly lower in healthy controls (G1) compared to G2 patients at TP0 and TP1 and significantly higher in G2 patients at TP0 compared to TP1. Therefore, all these periodontal indices significantly decreased after NSPT (Table 1).

The full-mouth mean reduction observed after the NSPT was 0.7 mm, and the mean clinical attachment gain (CAL-gain) was 0.6 mm, whereas in deeper pockets (≥5 mm), a mean reduction in pocket depth of 2.4 mm was observed.

The NSPT therapy was effective in reducing most of the periodontal pockets; the PPD reduction amount resulted directly correlated to the baseline (TP0) PPD, and on the contrary, the pocket closure was more frequent in shallower than deeper pockets. However, 117 pockets ≥ 5 mm, 61.58% of the overall pockets with PPD ≥ 5 mm at TP0 in G2 patients, became shallower (<5 mm) or closed after NSPT. In addition, an overall proportion of pocket closure of about 70% was observed.

### 3.3. Matrix Metalloproteinase-8 Protein (MMP-8) Assay

**Figure 2:**
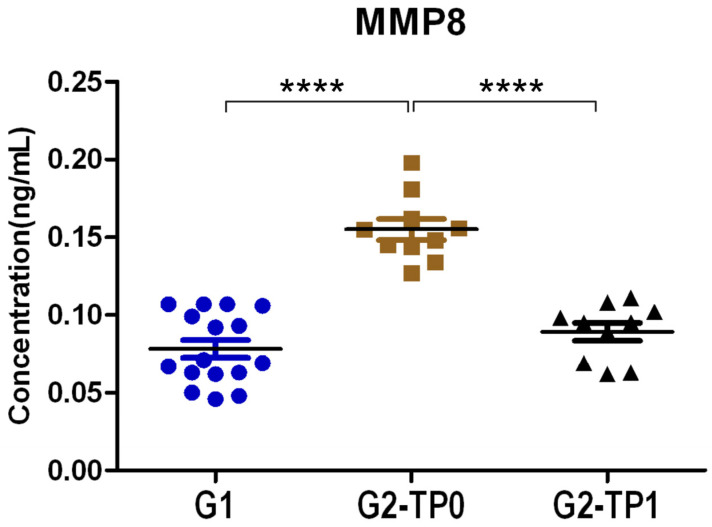
MMP-8 concentrations (ng/mL) in saliva of healthy controls (G1), periodontitis patients at baseline (G2-TP0), and periodontitis patients after NSPT therapy (G2-TP1) as measured by ELISA. Dots are individual values; bars represent mean ± SEM. **** = *p* < 0.0001.

Levels of MMP-8, one of the molecules mostly considered to obtain an objective diagnosis of PRD, were first measured (Figure 2). In all the groups considered, the MMP-8 concentration was quite low, as it never reached 0.2 pg/mL in any sample tested. G1 patients showed the lowest MMP-8 levels with a mean concentration of 0.08 ± 0.02 ng/mL. G2-TP0 had the greatest concentration of MMP-8, with an average of 0.16 ± 0.02 ng/mL, which was roughly double the concentration observed in healthy controls (G1). After PRD therapy (TP1), the concentration of MMP-8 in G2 patients was significantly reduced (0.09 ± 0.02 ng/mL) (Figure 2).

Statistical analysis revealed significant differences in the mean MMP-8 levels between G1 and G2 patients at TP0 (*p* < 0.001), as well as between G2 patients at TP0 and TP1 (*p* = 0.006). However, no significant difference was observed between G1 and G2 patients at TP1.

**Figure 3:**
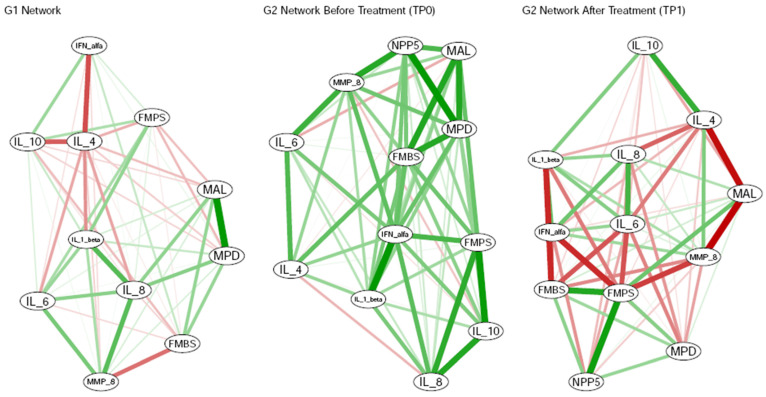
Spearman correlation analysis: Green interconnection lines indicate positive correlations, while red lines indicate negative correlations. The thickness of each line is proportional to the value of the Spearman’s correlation coefficient. Legend: TP = time point. IL = interleukin. IFN = interferon. MMP-8 = salivary matrix metalloproteinase-8. MAL = mean attachment level. MPD = mean probing depth. NPP5 = number of pockets with PPD ≥ 5 mm per patient. FMPS = full-mouth plaque score. FMBS = full-mouth bleeding score.

Furthermore, correlation analysis showed a significant negative correlation between MMP-8 and FMBS for G1 individuals (*ρ* = −0.55; *p* = 0.027) and between MMP-8 and MAL for G2 patients at TP1 (*ρ* = −0.777; *p* = 0.008). In contrast, a significant positive correlation was found between MMP-8 and NPP5 for G2 patients at TP1 (*ρ* = 0.699; *p* = 0.024) (Figure 3).

### 3.4. Cytokine Immunological Assay

We then measured the salivary concentration of an array of pro- and anti-inflammatory cytokines in the same samples to determine if PRD patients were characterized by an oral pro-inflammatory profile and if treatment contributed to resolving inflammation.

**Figure 4:**
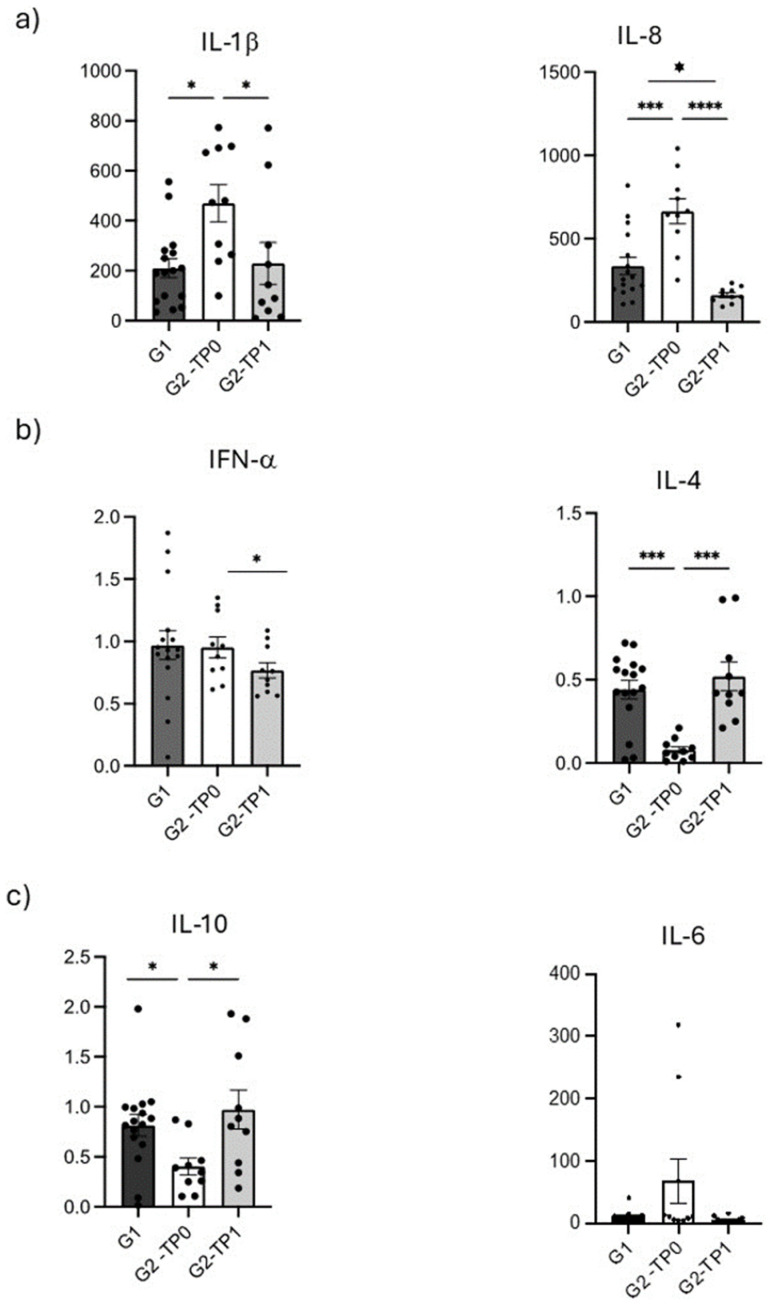
Cytokine concentrations (pg/mL) of (**a**) pro-inflammatory cytokines IL-1β and IL-8; (**b**) anti-inflammatory cytokines IL-4 and IL-10; (**c**) pleomorphic cytokines (pro- and anti-inflammatory cytokines) IL-6 and, in unusual cases, IFN-α measured in saliva of healthy controls (G1), periodontitis patients at baseline (G2-TP0) and periodontitis patients after periodontal treatment (G2-TP1). Dots are individual values; bars represent mean ± SEM. **** = *p* < 0.0001; *** = *p* < 0.001; ** = *p* < 0.01; * = *p* < 0.05.

Regarding pro-inflammatory molecules, IL-1β levels were significantly higher in G2 patients before treatment (TP0) (469.7 ± 234.1 pg/mL) compared to healthy controls (G1) (194.5 ± 157.8 pg/mL) (Figure 4a, left, and Table 2; *p*-value = 0.003). After treatment (TP1), G2 patients showed a significant reduction in IL-1β concentration, which, however, remained higher than the concentration observed in healthy controls (229.3 ± 265.7 pg/mL). IL-1β levels in G2 patients were significantly lower at TP1 compared to TP0 (*p* = 0.006), and the difference between G2 patients at TP1 and healthy controls was not statistically significant. Correlation analysis revealed a significant negative correlation between IL-1β and FMBS in G2 patients at TP1 (*ρ* = −0.66; *p* = 0.038).

**Figure 5:**
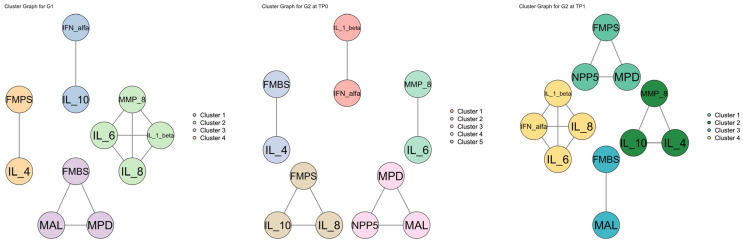
Cluster aggregation analysis for G1, G2 at TP0, and G2 at TP1: Interconnection lines indicate correlations among biomarkers and clinical indices. The different colors of the ellipsoidal shapes indicate the different clusters. Legend: TP = time point. IL = interleukin. IFN = interferon. MMP-8 = salivary matrix metalloproteinase-8. MAL = mean attachment level. MPD = mean probing depth. NPP5 = number of pockets with PPD ≥ 5 mm per patient. FMPS = full-mouth plaque score. FMBS = full-mouth bleeding score.

IL-8 followed a similar pattern to IL-1β. In G2 patients at TP0, IL-8 concentrations (664.2 ± 236.5 pg/mL) were significantly higher than those in healthy controls (336.1 ± 205.3 pg/mL; Wilcoxon rank-sum test, *p*-value = 0.002). After treatment, IL-8 levels significantly decreased in G2 patients compared to before the treatment (162.1 ± 45.6 pg/mL; *p*-value = 0.006). Healthy controls showed IL-8 levels significantly higher than G2 patients at TP1 (Figure 4a, right, and Table 2; *p*-value = 0.005). Correlation analysis revealed a significant positive correlation between IL-8 and FMPS in G2 patients at TP0 (*ρ* = 0.673; *p*-value = 0.033). Additionally, linear regression (LR) analysis revealed a significant linear relationship between IL-8 and FMPS in G1: a one-unit increase in IL-8 (pg/mL) was associated with an expected increase of 1.337 units in the mean value of FMPS (*p*-value = 0.025). Finally, cluster analysis for G2 patients at TP0 grouped IL-8, FMPS, and IL-10 into a common cluster (Figure 5). This finding provides preliminary statistical evidence of a shared pattern between the two cytokines and FMPS.

IFN-α was detected at low concentrations across all groups. Therapy significantly reduced IFN-α levels in G2 patients from TP0 (0.95 ± 0.27 pg/mL) to TP1 (0.77 ± 0.19 pg/mL) (Figure 4b, left, and Table 2; Wilcoxon signed-rank *p*-value = 0.014). In G1 patients, IFN-α concentration was 0.94 ± 0.48 pg/mL—slightly lower than in G2 patients at TP0—but no statistically significant differences were observed between healthy controls and G2 patients at either TP0 or TP1. In G2 patients at TP1, a significant negative correlation was found between IFN-α and FMPS (*ρ* = −0.648; *p*-value = 0.043). Neither cluster analysis nor LR provided statistically meaningful insights into the behavior of this cytokine.

Conversely, anti-inflammatory cytokines followed an opposite trend (Figure 3). IL-4 levels were generally low (always below 1 pg/mL) and significantly higher in G2 patients at TP1 (0.52 ± 0.27 pg/mL) with respect to G2 patients at TP0 (0.08 ± 0.06 pg/mL) (Figure 4b, right, and Table 2; Wilcoxon signed-rank *p*-value = 0.006). After treatment, IL-4 concentration rose significantly and reached values higher than those of healthy controls (0.44 ± 0.22 pg/mL). A significant statistical difference in the levels of IL-4 was detected between G1 and G2 at TP0 (*p*-value = 0.001), but no significant differences were observed between healthy controls and G2 patients at TP1. Correlation analysis revealed a significant negative correlation between IL-4 and MAL for G2 patients at TP1 (*ρ* = 0.737; *p*-value = 0.014). Finally, cluster analysis for G2 patients at TP0 grouped IL-4 and FMBS into a common cluster, whereas IL-4 was grouped with FMPS for G1 (Figure 5).

Similarly, IL-10 showed concentrations never higher than 2 pg/mL in any condition tested. G2 patients at TP0 (before treatment) exhibited IL-10 salivary concentration (0.97 ± 0.62 pg/mL) significantly lower than control G1 (0.81 ± 0.44 pg/mL; *p*-value = 0.014) and G2 patients at TP1 (*p*-value = 0.011). Treatment of PRD determined an increase in the concentration of the cytokine in G2 patients at TP1 (0.97 ± 0.62 pg/mL), who showed IL-10 concentrations higher than the control G1, but without statistical significance (Figure 4c, left, and Table 2). Correlation analysis detected a significant positive correlation between IL-10 and FMPS for G2 patients at TP0 (*ρ* = 0.794; *p*-value = 0.006). Additionally, linear regression analysis revealed a significant linear relationship between IL-10 and FMPS in G1: a one-unit increase in IL-10 (pg/mL) was associated with an expected increase of 0.915 units in the mean value of FMPS (*p*-value = 0.035).

We finally measured IL-6, which can have both pro- and anti- inflammatory effects (Figure 4c, right, and Table 2). IL-6 showed a trend comparable to that of IL-1β and IL-8. Indeed, IL-6 levels were higher in G2 patients at TP0 (67.73 ± 113.32 pg/mL) than controls (11.05 ± 8.71 pg/mL) and diminished to levels lower than those of controls at TP1 (6.50 ± 4.47 pg). Wilcoxon signed-rank test detected a significant difference in the levels of IL-6 for G2 at TP0 vs. TP1 (*p*-value = 0.011), but no significant differences between the patient groups (G1 and G2) at either time point of assessment (TP0 and TP1). Spearman’s correlation test or cluster analysis did not uncover any significant relationships between IL-6 and the clinical indices (Table S1).

### 3.5. Logistic Regression

Diagnostic testing using the “detectseparation” package in R confirmed complete separation, indicating that one or more biomarkers perfectly distinguished between outcome groups.

In the comparison between healthy controls (G1) and PRD cases (G2 at TP0), four cytokine biomarkers showed statistically significant associations with disease status: IL-1β (β = 0.0067, *p* = 0.0108), IL-8 (β = 0.0062, *p* = 0.0087), IL-4 (β = −11.1641, *p* = 0.0118), and IL-10 (β = −3.5171, *p* = 0.0210). These coefficients represent the change in log-odds per 1 pg/mL increase in biomarker level. IL-1β was associated with a 0.67% increase in the odds of PRD [OR = 1.0067 = exp(0.0067)], and IL-8 was associated with a 0.62% increase in the odds of PRD [OR = 1.0062 = exp(0.0062)].

IL-4 showed a protective association reducing the odds of PRD; per 1 pg/mL increase in biomarker level, the odds of disease reduce by ≈99.99% [OR = 0.0000142 = exp(−11.164)]. Similar interpretations apply to IL-10; per 1 pg/mL increase in biomarker level, the odds of disease reduce by ≈97% [OR = 0.030 = exp(−3.517)] (Table 3).

**Figure 6:**
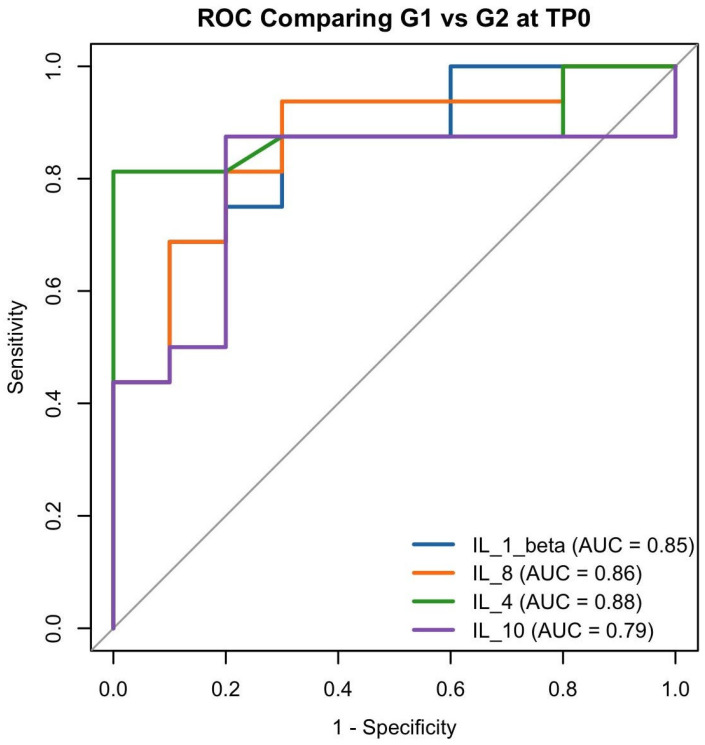
G1 vs. G2 at TP0 ROC curves for IL-1β, IL-8, IL-4, and IL-10 distinguishing healthy controls (G1) from untreated periodontitis patients (G2 at TP0). AUC values indicate each biomarker’s individual classification performance.

Four separate ROC curves were generated, one for each of the four biomarkers that showed a statistically significant association in univariate logistic regression. Each ROC curve in Figure 6 represents the diagnostic ability of a single biomarker to distinguish between periodontitis cases (G2 at TP0) and healthy controls (G1), based on the predicted probabilities from its corresponding univariate model (Figure 6).

**Figure 7:**
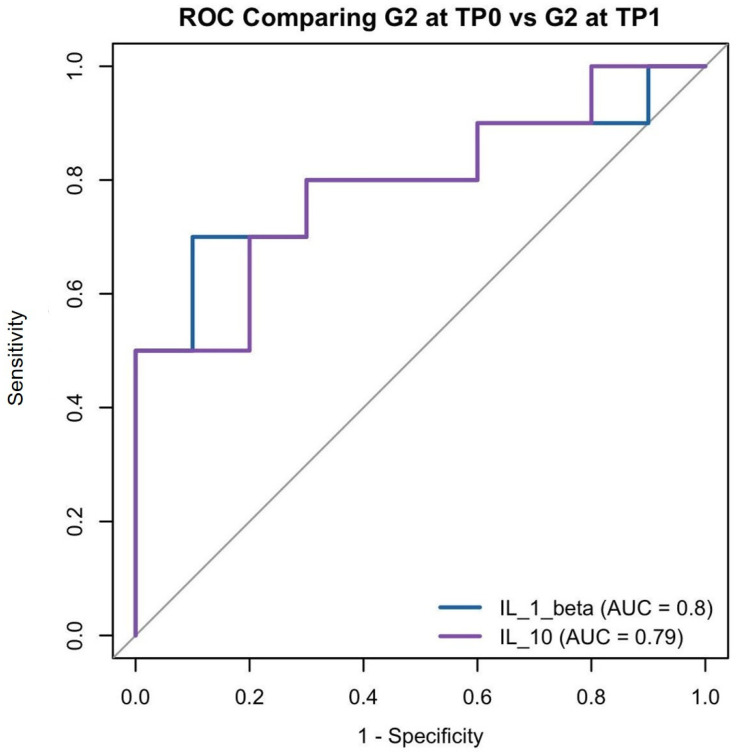
G2 at TP0 vs. G2 at TP1 ROC curves for IL-1β and IL-10 distinguishing untreated periodontitis patients (G2 at TP0) from treated periodontitis patients (G2 at TP1). AUC values indicate each biomarker’s individual classification performance.

We then compared PRD patients before (G2 at TP0) and after treatment (G2 at TP1). Similar separation issues arose in the multivariate model, so we again relied on univariate analyses. IL-1β (β = −0.0038, *p* = 0.059) and IL-10 (β = 3.289, *p* = 0.0511) did not show statistical significance, but a statistical borderline (Table 4). As a trend, these results could be biologically relevant. ROC curves (Figure 7) summarize their ability to distinguish treatment status.

**Figure 8:**
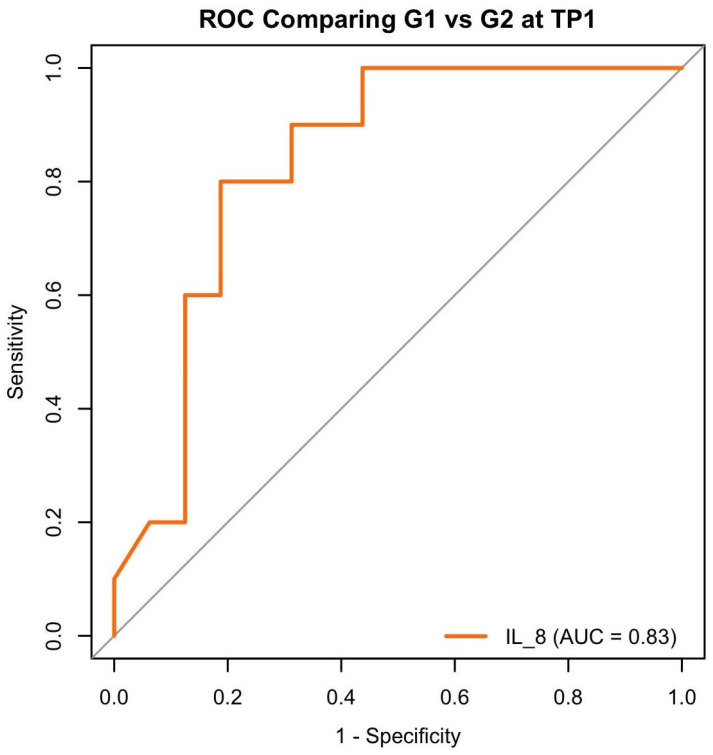
G1 vs. G2 at TP1 ROC curves for IL-8 distinguishing healthy controls (G1) from treated periodontitis patients (G2 at TP1). AUC values indicate biomarker’s individual classification performance.

Finally, we compared healthy controls (G1) with treated PRD patients (G2 at TP1). Multivariate analysis showed no signs of separation but did not identify any significant predictors. In univariate models, only IL-8 was statistically significant (β = −0.0175, *p* = 0.0472), with higher levels being associated with lower odds of being in the treated group (OR ≈ 0.9896), possibly suggesting possible changes in inflammatory response following therapy (Table 5 and Figure 8).

Interestingly, MMP-8 showed complete separation in univariate models for both G1 vs. G2 at TP0 and G2 at TP0 vs. TP1. Biomarker values perfectly or nearly perfectly distinguished between groups, making the ROC curve somewhat pleonastic. This finding highlights the potential of MMP-8 as a strong discriminator of PRD status and treatment response in this dataset.

## 4. Discussion

In this study, we simultaneously considered the main periodontal clinical parameters related to the full mouth (FMPS, FMBS, MAL, MPD, and NPP5), alongside a biomarker molecular network. This approach allows for the integration of validated clinical diagnostic and prognostic processes related to objectively measured significant molecular markers. The decision to use clinical indices that reflected the condition of the entire mouth was also influenced by the type of periodontal therapy administered—non-surgical periodontal therapy (NSPT), which is not aimed at treating a specific defect or a particular type of periodontal defect but rather addresses the patient’s entire periodontal system. The molecular analyses aimed to identify early connective tissue damage associated with immune responses, inflammation initiation, and resolution mechanisms, enabling a comprehensive evaluation of significant molecular markers of periodontitis (PRD) in relation to clinically validated diagnoses. The cytokine network specific to PRD likely precedes periodontal damage, allowing for timely interventions in periodontal therapy to prevent disease progression.

By comparing patients with complete periodontal health (G1) to those suffering from severe or advanced PRD (G2) [34,35], we can enhance the study’s capacity to identify significant molecular differences between periodontal health and more advanced stages of the disease.

NSPT was able to reduce overall probing depths, particularly in stratified probing depths. The reduction was significantly greater in teeth with higher probing pocket depth (PPD) at baseline. Conversely, pocket closure occurred more frequently in shallower pockets than in deeper ones. NSPT proved to be an effective treatment for reducing inflammation, probing pocket depth, and the number of diseased sites in patients affected by periodontitis. Therefore, it appears that well-executed NSPT may limit the need for additional or alternative treatment approaches, which could be more invasive and entail higher costs. Additionally, this study demonstrated that in advanced PRD cases, NSPT therapy was more effective in reducing PPD, although complete disease resolution, as measured by pocket closure, was less likely.

The FMBS is considered a clinical index of periodontal inflammation, directly related to the presence and amount of subgingival deposits and the periodontal inflamed surface area (PISA) [55,56,57]. The FMBS serves as a useful prognostic indicator in clinical diagnosis, particularly during the periodontal maintenance phase [58]. Moreover, the correlation of FMBS with MPD (and NPP5) provides important insights into biological phenotypes that can guide diagnoses and treatments in any therapeutic phase. The extent of FMBS and MPD scores was associated with distinct inflammatory, immune, and microbial characteristics, leading to separate biological phenotypes [55,59,60].

The clinical parameters measured at TP0 and TP1 aligned with the molecular analyses as a rule. Salivary harvesting has gained significance in clinical diagnostics compared to other biological fluids, primarily because saliva is easily accessible and its collection is safe and non-invasive for patients [61].

MMP-8 has recently emerged as a key molecule in the pursuit of an objective diagnosis for PRD [12,14,16,62]. MMP-8 is primarily involved in the non-specific breakdown of periodontal collagen and plays a complex role in immune modulation [36,37,38]. It is primarily secreted by neutrophils (closely associated with the “hyperactivated” neutrophil phenotype) and plays an important regulatory role in both acute and chronic inflammation [18,63,64]. Present at the initial stages of inflammatory reactions, MMP-8 can influence their outcomes and is upregulated in tissue remodeling processes (Table 2) [65,66].

In this study, the MMP-8 analysis revealed greater concentrations in G2 at TP0, lower levels in G1, and intermediate levels in G2 at TP1, with significant differences observed between G2 at TP0 and both G1 and G2 at TP1, but not between G1 and G2 at TP1. The higher levels of MMP-8 in the population affected by severe/advanced PRD at TP0 are likely related to the cleavage of periodontal collagen and the inflammatory phase. G2 patients at TP1 exhibited, on average, a certain number of periodontal pockets and lower MMP-8 levels compared to G2 patients at TP0. Interestingly, Spearman’s rank correlation, which analyzes the relationships between clinical indices and MMP-8, identified correlations in G1, in G2 at TP1, and in G2 at TP0.

These results, along with the greater dosage of MMP-8 detected in the saliva of G2 patients at TP1 compared to patients free of PRD in G1 (without statistical significance), highlight a pathogenic aspect of PRD and suggest a favorable effect of non-surgical periodontal therapy (NSPT). It is conceivable that, although collagen cleavage and inflammation were substantially reduced following NSPT, they were not completely abolished, as observed in G1 patients.

In logistic analysis, MMP-8 showed complete separation in univariate models for both G1 vs. G2 at TP0 and G2 at TP0 vs. TP1. However, despite these very impressive results, the complexity of its biological functions and its lack of strict specificity for the PRD limit its utility as a standalone diagnostic tool and highlight the need for further research.

Similar to MMP-8, IL-6, IFN-α, IL-1β, IL-8, IL-4, and IL-10 are also considered eligible biomarkers that can be routinely utilized for chair-side early diagnosis of tissue inflammatory conditions and PRD [67,68]. IL-6 and IFN-α are strongly associated with both local and systemic inflammatory processes [69,70,71,72,73,74,75] (Table 2).

IFN-α contains a mixture of several proteins with partially overlapping structural, functional, and serological properties. This cytokine plays a substantial role in inducing a hyperactive phenotype in neutrophils, promoting oxidation through the reactive oxygen species (ROS) production, and contributing to the chronic inflammation observed in periodontal tissues [67,71,75,76,77,78,79] (Table 2). Following successful treatment for periodontitis, plasma IFN-α levels and neutrophil hyperactivity decreased to levels comparable with those of controls [67].

In this study, IL-6 and IFN-α showed greater concentrations in G2 patients at TP0, lower concentrations in G2 patients at TP1, and intermediate levels in G1 patients. IFN-α and IL-6 showed a significant difference only between G2 patients before and after treatment. IFN-α resulted in a correlation with FMPS in G2 at TP1.

IL-1β and IL-8 are considered potent pro-inflammatory interleukins (ILs) [23,80] and potential therapeutic targets for PRD [10,68,81]. Furthermore, IL-1β and IL-8 interact with each other, with IL-1β inducing IL-8 synthesis (Table 2) [82]. They recruit neutrophils in response to bacterial challenges, promote angiogenesis, and are involved in various cellular activities, including cell proliferation, differentiation, apoptosis, and initiation of the oxidative burst [23,80,82,83]. Their role is strategic in infection control [84,85]. Both IL-1β and IL-8 are also considered candidates for chair-side early diagnosis of PRD [55,68,86].

In this study, both IL-1β and IL-8 showed greater concentrations in G2 patients at TP0, with statistically significant differences between G2 patients at TP0 and TP1. IL-1β exhibited lower concentrations in G1 patients, while IL-8 showed lower concentrations in G2 patients at TP1. No statistical significance was found between G1 and G2 patients at TP1 for IL-1β. In G2 at TP0, IL-8 was found to cluster with FMPS and IL-10. Notably, IL-10 consistently grouped with IL-8, showed a positive correlation, and exhibited a linear relationship with FMPS in G1. However, IL-1β resulted in inverse correlation with FMBS in G2 at TP1. Additionally, logistic analysis showed that an increase in IL-8 predicted a decreased risk of disease, with an odds ratio favoring G1 over G2 at TP1, suggesting changes in the inflammatory response following therapy in PRD patients.

Logistic regression confirmed the disease risk prediction for both IL-1β and IL-8 when comparing G1 vs. G2 at TP0. A decrease in IL-1β predicted a good outcome of periodontal therapy as a trend, without reaching statistical significance (borderline significance), when comparing G2 at TP0 vs. G2 at TP1.

IL-4 and IL-10 are recognized as major anti-inflammatory cytokines [75]. Low blood levels of IL-10 and IL-4 may contribute to chronic inflammatory pain [87]. Among anti-inflammatory cytokines, IL-10 is particularly potent, repressing the expression of pro-inflammatory cytokines and regulating endogenous anti-cytokines. It can counteract the production and function of pro-inflammatory cytokines at multiple levels (Table 2) [75].

In this study, both IL-4 and IL-10 exhibited higher concentrations in G2 patients at TP1 and lower concentrations in G2 patients at TP0, with G1 patients falling in between. Statistically significant differences were observed between G1 and G2 at TP0, as well as between G2 at TP0 and TP. Additionally, these results correlated with the periodontal indices considered, showing an inverse relationship between IL-4 and IL-10 levels and the clinical features of PRD. Treatment for G2 patients significantly increased anti-inflammatory IL levels, while PRD-free G1 patients showed significantly higher concentrations than G2 patients at TP0. IL-4 clustered with FMBS in G2 at TP0 and with FMPS in G1. IL-4 was inversely correlated with MAL in G2 at TP1. The logistic regression predicted a decrease in disease risk with the increase in both the ILs when comparing G1 vs. G2 at TP0, and the IL-10 increase predicted a good outcome of periodontal therapy when comparing G2 at TP0 vs. G2 at TP1 as a trend, with borderline statistical significance.

All the pro-inflammatory cytokines considered (IL-1β, IL-8, IL-6, IFN-α) showed greater concentrations in G2 patients at TP0, while the anti-inflammatory cytokines (IL-4 and IL-10) exhibited lower concentrations in G2 patients at TP0. Almost all the pro-inflammatory cytokines (IL-8, IL-6, and IFN-α, but not IL-1β) displayed lower concentrations in G2 patients at TP1, whereas the anti-inflammatory cytokines (IL-4 and IL-10) showed greater concentrations in G2 patients at TP1. The cytokine concentrations (IL-1β, IL-8, IFN-α, IL-4, and IL-10) and MMP-8 levels between G2 patients at TP0 and G2 patients after NSPT at TP1 were mostly significantly different. Additionally, G1 and G2 patients at TP0 often exhibited significant differences in cytokine concentrations (IL-1β, IL-8, IL-4, and IL-10) and MMP-8 levels, while G1 and G2 patients after treatment at TP1 showed a significant difference in IL-8 concentrations.

Cytokines, including interleukins (ILs), play a crucial role in regulating inflammation, which is essential for eliminating pathogenic bacteria and stimulating the regeneration of affected tissues [88]. Since microbiota challenges and inflammation precede periodontal damage [30,32,89,90], it is conceivable that the selected cytokines could predict periodontal damage before it occurs, thereby enabling an early diagnosis of PRD, before periodontal damage has taken place, making the clinical diagnosis occur later than the actual onset of damage.

Despite the small sample size, the biomarker network examined has proven effective in confirming the diagnosis of severe PRD and in monitoring the effects of NSPT in a minimally invasive manner. Therefore, it represents a promising approach for further studies aimed at achieving more consolidated results in this area.

Both MMP-8 and the cytokine network, particularly IL-1β, IL-4, IL-8, and IL-10, are associated with PRD. However, neither MMP-8 nor the single cytokines alone are strictly specific to the condition. Data and ROC curves demonstrated a close relationship between these molecules and periodontal health, PRD, and treated PRD.

An imbalance between pro-inflammatory and anti-inflammatory mediators during acute inflammatory challenges plays a central role in the development and progression of both infectious diseases (such as PRD) and chronic inflammation [91]. Therefore, in a healthy (G1) or treated patient (G2-TP1), the presence of inflammatory cytokines such as IL-8 could represent a defense mechanism against the challenge posed by microbiota, rather than a precipitating factor leading to chronic inflammation. In addition, in G2 at TP0, their correlations with clinical indices are positive; an increase in FMPS, FMBS, or NPP5 predicted an increase in MMP-8 and IL-8 levels. In contrast, in G1 or in G2 at TP1 (i.e., after periodontal therapy), the associations between biomarkers and clinical indices were predominantly negative. This study suggests that the molecular network under investigation may reflect two distinct behavioral patterns associated with PRD: one linked to periodontal health (G1) or post-treatment conditions (G2-TP1) and another characteristic of patients with active PRD. However, the current data primarily support the utility of individual biomarkers and the feasibility of the network approach, rather than a fully validated network model.

The molecular physiological defense mechanisms and tissue homeostasis undergo significant changes, becoming part of the disease-specific damage mechanisms. This shift goes beyond the normal pleomorphic biological behavior of cytokines.

## 5. Strengths and Limitations of the Study

In this study, 61.5% of the patients were systemically healthy and unaffected by PRD or gingivitis. This represents an important sample for a study aiming to discriminate among patients with PRD (G2 at TP0), those who have been treated for PRD (G2 at TP1), and patients without PRD (G1), that is, those in complete periodontal health [34,35,92]. The inclusion of a G1 control group composed entirely of systemically healthy individuals with full periodontal health is not very frequent in PRD studies but offers significant advantages. Most notably, it allows for more effective comparisons between diseased and ideally healthy subjects, thereby enhancing the ability to distinguish clinical and biomarker differences.

In this study, in G2 patients at TP0 (untreated periodontitis patients), a lack of anti-inflammatory cytokines was found. In addition, directly in periodontal pocket tissues, a significant deficiency in the pro-resolving inflammation protein network was observed [6,93,94]. Our results confirm what has already been observed in periodontal pockets in relation to the pathogenesis of periodontitis, which appears to be associated with a pathological management of inflammation, including a deficiency in the active mechanisms responsible for its resolution [95,96].

On the contrary, a significant limitation of the study is the small sample size. Selecting compliant patients who were systemically healthy, non-smokers, and affected only by PRD (to avoid confounding variables) proved challenging, as did finding completely healthy control subjects.

In this preliminary study, we selected a network of biomarkers that could each be individually associated with PRD and that exhibited the characteristic of being relatively stable over time in healthy adult subjects.

None of the individual biomarkers, however, was strictly specific to PRD; therefore, the idea of considering them as a network could reduce the non-specificity resulting from evaluating any single marker in isolation. Moreover, the variation of these biomarkers with age was considerably less significant than the variation that could occur in relation to PRD.

This study compares a group of healthy subjects with a group of patients; however, a limitation arises from the limited knowledge, particularly regarding the long-term stability of cytokine concentrations in healthy individuals. There is a clear need for studies evaluating the stability of biomarkers over the medium and long term, which remains an open question.

Nevertheless, the results obtained provide a valuable starting point for future research involving a larger sample size.

A future research roadmap may build upon our preliminary findings but will require larger sample sizes to overcome current statistical limitations, achieve more comprehensive results, and allow for a more refined characterization of the molecular network in relation to its clinical utility. A larger sample size would address the current statistical limitations in several ways. With more observations, estimates of both within-group and between-group variability become more reliable, reducing the likelihood that any single biomarker perfectly separates outcome classes. This, in turn, would allow for the simultaneous evaluation of multiple biomarkers via multivariate analysis, providing stable estimates of coefficients, confidence intervals, and potential interactions within the molecular network. We could then more accurately determine the predictive power of the biomarker network for distinguishing disease states and monitoring treatment response.

## 6. Conclusions

Within the limitations of this study, we conclude the following:

- The NSPT produces clinically relevant effects, reducing the need for further, more invasive therapies.
- Molecular analysis, using validated procedures, provides objective and useful measurements.
- MMP-8 levels could reliably help differentiate between subjects with a healthy periodontium and those affected by severe PRD.
- The analysis of the biomarkers under consideration is at least a useful aid in differentiating between individuals with a healthy periodontium and patients with PRD. Assuming that changes in the biomarkers considered can be detected before periodontal damage occurs, this approach may serve as a valid starting point for effective early diagnosis of the disease, before tissue destruction becomes established. Moreover, the molecular network studied—including MMP-8—appears to have potential for monitoring the response to PRD therapy and provides a promising basis for future research. MMP-8, IL-1β, IL-4, IL-8, and IL-10 show the strongest statistical associations with clinical indices in both healthy and diseased states.
- Statistical evaluations reveal a clear distinction between the untreated PRD phase (G2 at TP0), the treated PRD phase (G2 at TP1), and periodontal health (G1), in terms of the behavior of clinical indices relative to the investigated biomarkers. These findings suggest that individuals with periodontal health or treated PRD could manage inflammation in a significantly different manner compared to those with untreated PRD.

## Figures and Tables

**Table 1:** Main features of G1 (controls) and G2 patients at TP0 and TP1.

G1		G2	
	TP0	TP0	TP1
N (females)	16 (8)	10 (6)	
Age (years)	24.9 ± 4.53	52.2 ± 8.28 *	
FMPS (%)	8.12 ± 6.03	41.10 ± 22.20	20.72 ± 9.68 *°
FMBS (%)	3.62 ± 3.12	33.80 ± 15.34	16.90 ± 10.82 *°
MPD (mm)	1.24 ± 0.15	2.63 ± 0.65	1.94 ± 0.42 *°
MAL (mm)	0	3.56 ± 0.75	2.95 ± 0.67 *°
NPP5	0	19.00 ± 12.76	7.30 ± 11.12 *°

Data are expressed as mean ± SD. At TP0, G2 parameters significantly different from G1 are in bold. * significant difference of G2 patients at TP1 vs. healthy control. ° significant difference of G2 patients at TP0 vs. TP1. MPD = mean probing depth; MAL = mean attachment level; NPP5 = number of periodontal pockets.

**Table 2:** MMP-8 and cytokines: expression in periodontitis patients at TP0, functions, and interactions.

Biomarker	Expression in Periodontitis	Function	Interaction
MMP-8	Upregulated	– Non-specific connective tissue cleavage; – Connective tissue remodeling; – Promotes inflammation by enhancing leukocyte infiltration; – Resolves inflammation by degrading pro-inflammatory signals; – Overexpression is associated with chronic inflammation.	– IL-1β strongly induces MMP-8 expression; – IL-6 promotes MMP-8 expression in an inflammatory setting, depending on the presence of neutrophils.
IL-1β	Upregulated	– Pro-inflammatory; – Bone resorption; – Non-specific connective cleavage; – Transition from acute to chronic inflammation.	– Stimulates IL-8 production. – Activates neutrophils; – Stimulates osteoclastogenesis; – Stimulates MMP (matrix metalloproteinase) production; – Inhibited by IL-4 and IL-10.
IL-8	Upregulated	– Pro-inflammatory; – Neutrophil recruitment; – Transition from acute to chronic inflammation.	– Amplified by IL-β1; – Enhanced by IL-6; – Inhibited by IL-4/and IL-10.
IL-6	Upregulated	– Exhibits both pro-inflammatory and anti-inflammatory effects (depending on the context of its production); – Induces acute-phase protein production; – Causes non-specific connective tissue cleavage.	– Activates neutrophils; – Stimulates osteoclast differentiation; – Stimulates MMP production, and IL-1β promotes IL-6 production; – Downregulated by IL-4 and IL-10.
IFN-α	Upregulated	– Exhibits both pro-inflammatory and anti-inflammatory effects; – Involved in antiviral immune responses; – Exacerbates the inflammatory response; – Induces chronic inflammation; – Induces a hyperactive phenotype in neutrophils; – Promotes ROS production; – Contributes to the induction of chronic inflammation.	– The expression of IFN-α is enhanced by IL-1β; – IL-10 antagonizes IFN-α inflammatory activity; – IFN-α production is suppressed by IL-10; – IL-4 inhibits IFN-α expression.
IL-4	Downregulated	– Anti-inflammatory; promotes Th2.	– Suppresses IL-1β and IL-8.
IL-10	Downregulated or insufficient	– Anti-inflammatory; suppresses Th1.	– Suppresses IL-1β and IL-8; induced by IL-6.

Legend: TP = time point. IL = interleukin. IFN = interferon. MMP-8 = salivary matrix metalloproteinase-8. ROS = reactive oxygen species. Th1 = T helper cell 1. Th2 = T helper cell 2.

**Table 3:** 43vs. PRD patients (G2-TP0).

Biomarker	β (Coeff.)	St. Error	*p*-Value	OR (exp(β))	95% IC OR	Effect % on Odds	Biologic Significance
IL-1-β	0.0067	0.0026	0.0108	1.0067	[1.0016–1.0119]	↑ +0.67%	Increased Risk
IL-8	0.0062	0.0024	0.0087	1.0062	[1.0015–1.0110]	↑ +0.62%	Increased Risk
IL-4	−11.1641	4.4322	0.0118	0.0000142	[6.19e−08–0.0334]	↓ −99.99%	Reduced Risk
IL-10	−3.5171	1.5243	0.0210	0.030	[0.0021–0.4416]	↓ –97%	Reduced Risk

↑ indicates increased risk; ↓ indicates reduced risk.

**Table 4:** State of illness; patients with untreated PRD (G2-TP0) vs. treated PRD (PRD G2- TP1).

Biomarker	β (Coeff.)	St. Error	*p*-Value	OR (exp(β))	95% IC OR	Effect % on Odds	Biologic Significance
IL-1-β	−0.0038	0.0020	0.059	0.9962	[0.9923–1.0001]	↑ +0.38%	Increased risk
IL-10	3.289	1.687	0.0511	26.81	[2.67–268.33]	↑ +2581%	Reduced Risk

↑ indicates increased risk.

**Table 5:** State of illness; subjects without PRD (G1) vs. treated PRD (PRD G2- TP1).

Biomarker	β (Coeff.)	St. Error	*p*-Value	OR (exp(β))	95% IC OR	Effect % on Odds	Biologic Significance
IL-8	−0.0175	0.0089	0.0472	0.9896	[0.9657–0.9999]	↑ 1.74%	Reduced Risk

↑ indicates increased risk.

## Data Availability

The data presented in this study are available on reasonable request from the corresponding author. The data are not publicly available due to privacy and ethical restrictions.

## Supplementary Materials

The following supporting information can be downloaded at https://www.mdpi.com/article/10.3390/dj13090436/s1. Table S1: Biomarker levels (mean ± SD) in the study groups. G0: healthy controls; G1: periodontitis at baseline (untreated); G2: periodontitis after periodontal therapy.

## Abbreviations

The following abbreviations are used in this manuscript:

AUC	Area Under the Curve
CAL	Clinical Attachment Level
DM	Diabetes Mellitus
FMBS	Full-Mouth Bleeding Score
FMPS	Full-Mouth Plaque Score
G1	Group 1: Healthy Controls
G2	Group 2: Patients with PRD
GCF	Gingival Crevicular Fluid
IFN-α	Interferon Alpha
IL	Interleukin
MAL	Mean Attachment Level
MMP-8	Matrix Metalloproteinase-8
MPD	Mean Probing Depth
NPP5	Number of Pockets with PPD ≥ 5 mm
NSPT	Non-Surgical Periodontal Therapy
OD	Optical Density
PPD	Probing Pocket Depth
PRD	Periodontitis
RA	Rheumatoid Arthritis
REC	Gingival Recession Depth
ROC	Receiver Operating Characteristic
SD	Standard Deviation
SE	Standard Error
TP0	Time Point 0 (Baseline)
TP1	Time Point 1 (Post-treatment Follow-Up)
