# Supplementary material for: Salivary Biomarker Analysis to Distinguish Between Health and Periodontitis Status: A Preliminary Study
Source: Dent J (Basel). 2025 Sep 22;13(9):436. doi: 10.3390/dj13090436 (PMC12468743; doi:10.3390/dj13090436)
Supplement: Supplementary file 1 [file dentistry-13-00436-s001.zip › dentistry-3824802-supplementary.pdf]

Supplementary

Supplementary Table S1. Biomarker levels (mean ± SD) in the study groups. G0: healthy controls; G1: periodontitis at baseline (untreated); G2: periodontitis after periodontal therapy.

**Biomarker**	**G1 (Controls)**	**G2 at T0 (Untreated PRD)**	**G2 at T1 (Treated PRD)**
IL-1β (pg/mL)	194.5 ± 157.8	469.7 ± 234.1	229.3 ± 265.7
IL-4 (pg/mL)	0.44 ± 0.22	0.08 ± 0.06	0.52 ± 0.27
IL-6 (pg/mL)	11.05 ± 8.71	67.73 ± 113.32	6.50 ± 4.47
IL-8 (pg/mL)	336.1 ± 205.3	664.2 ± 236.5	162.1 ± 45.6
IL-10 (pg/mL)	0.81 ± 0.44	0.55 ± 0.21	0.97 ± 0.62
MMP-8 (ng/mL)	0.08 ± 0.02	0.16 ± 0.02	0.09 ± 0.02
